# Epidemiological and clinical description of patients with oropharyngeal cancer treated in a public oncology referral hospital in Chile

**DOI:** 10.3332/ecancer.2024.1685

**Published:** 2024-03-26

**Authors:** V Felipe Carvajal, M Felipe Cardemil, Beatriz P Vásquez, Carolina E Oliva, Tamra A Barría, Maribel A Bruna, Leonor A Moyano, Felipe A Bustos, Paola A Muñoz, Cristóbal F Araya, Jorge E Oyarzún, Eduardo A Villa, Federico A Floriano, Alexis J del Rio, Sebastián R Indo, Enrique A Castellón, Héctor R Contreras

**Affiliations:** 1 Department of Radiotherapy, National Cancer Institute, Santiago 8380000, Chile; 2 Department of Radiotherapy, Hospital Base Valdivia, Los Ríos 5090145, Chile; 3 Department of Basic Clinical Oncology, School of Medicine, Universidad de Chile, Santiago 8380453, Chile; 4 Department of Otolaryngology, School of Medicine, Universidad de Chile, Santiago 8380453, Chile; 5 Department of Basic Clinical Oncovirology, School of Medicine, Universidad de Chile, Santiago 7591047, Chile; 6 Department of Otolaryngology, Clínica Las Condes, Región Metropolitana 7500922, Chile; 7 Department of Otolaryngology, Hospital del Salvador, Región Metropolitana 8380453, Chile; 8 Department of Anatomic Pathology, National Cancer Institute, Santiago 8380000, Chile; 9 Department of Head and Neck Surgery, National Cancer Institute, Santiago 8380000, Chile; 10 Centro de Investigación y Especialidades Médicas (CDIEM), Santiago 7500859, Chile; 11 Department of Radiotherapy, Hospital Regional de Talca, Talca 3460001, Chile; 12 Department of Head and Neck Surgery, Clínica Alemana, Región Metropolitana 7650568, Chile; 13 School of Dentistry, Universidad de Chile, Santiago 8380453, Chile; 14 School of Medicine, Universidad de Chile, Santiago 8380453, Chile; 15 Management Information Area, Medical Subdirectorate of Institutional Development, National Cancer Institute, Santiago 8380000, Chile; 16 School of Medical Technology, School of Medicine, Universidad de Chile, Santiago 8380453, Chile; 17 Department of Medical Technology, School of Medicine, Universidad de Chile, Santiago 8380453, Chile

**Keywords:** human papillomavirus, epidemiology, squamous cell carcinoma of head and neck, oropharyngeal neoplasms

## Abstract

**Introduction:**

The incidence of squamous carcinoma of the oropharynx (OPSCC) has presented an increase worldwide, a fact that occurs along with a phenomenon of epidemiological transition, whose pathogenesis is linked to human papilloma virus (HPV) in a significant part of the cases. Published evidence at the Latin American level is scarce. The present study aims to evaluate the epidemiological and clinical characteristics of patients with oropharyngeal cancer treated in a public oncology reference centre in Chile.

**Methodology:**

A cross-sectional study was carried out. Patients with histological confirmation of OPSCC aged 18 years or older, referred to the National Cancer Institute of Chile between 2012 and 2023 were included. The association with HPV was determined by immunohistochemistry for p16.

**Results:**

178 patients were analysed, most of them in locoregionally advanced stages involving the palatine tonsil. Seventy-seven percent were male, with a median age of 60 years. Sixty-seven percent of patients were positive for p16, with a progressive increase to 85% in the last 2 years of the study. The p16(+) patients were younger and had fewer classical risk factors. Primary treatment was radiotherapy in 94% of patients.

**Conclusion:**

The epidemiological profile of patients with OPSCC treated in a Chilean public oncology referral centre reflects the epidemiological transition observed in developed countries. This change justifies the need to adapt health policies and conduct research that considers the characteristics of this new epidemiological profile.

## Introduction

The incidence of squamous carcinoma of the oropharynx (OPSCC) has increased during the last few years worldwide according to data recorded mainly in North America and Europe [1–4]. In addition, an epidemiological transition phenomenon has been observed, currently affecting a younger group of patients, with a lower prevalence of classic risk factors such as alcohol and tobacco consumption, and whose pathogenic factor has been determined to be the human papilloma virus (HPV) in a significant number of cases [5–8]. This group has been called ‘the new head and neck cancer patient’ and would have a better oncologic prognosis [7]. Lechner *et al* [7]. This group in particular is more exposed to a decrease in their quality of life as a consequence of adverse effects, due to the therapy received at an earlier age and with a longer survival, so the number of patients exposed to live with potential adverse effects of any of the therapies performed is higher. For this reason, the outcome in terms of quality of life of new therapeutic strategies has been studied, incorporating advanced radiotherapy techniques and transoral surgical procedures in the management of early-stage patients [7]. In Latin America, the published evidence regarding this group of patients is scarce [9]. Oliveira *et al* [9]. The present study aims to describe the epidemiological and clinical characteristics of patients with oropharyngeal cancer treated in a public oncology referral hospital in Chile during the years 2012–2023.

## Methodology

**Figure 1.**
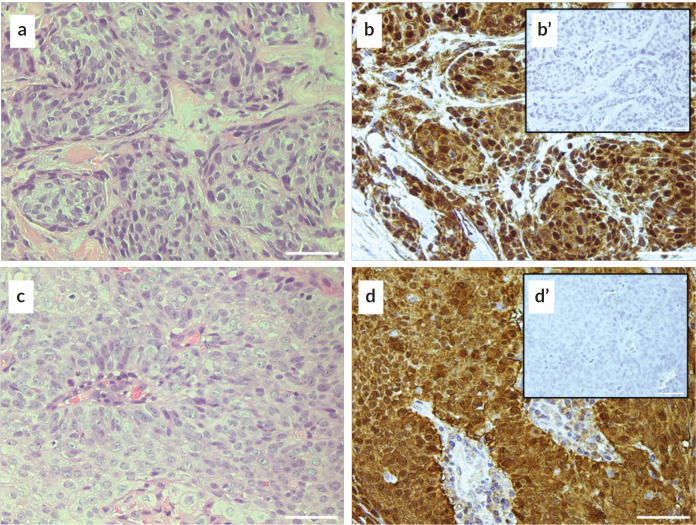
p16 identification in two representative samples of patients with oropharyngeal cancer. (a and b): histological sections stained with hematoxylin and eosin from two patient samples. (c and d): immunohistochemical identification of p16, contrast with hematoxylin. Both patients presenting more than 70% positivity for the marker. (c’ and d’): inserts showing the negative controls of IHQ p16. Bar: 50 m.

A retrospective cross-sectional study was performed. The protocol was approved by the research committee of the National Cancer Institute of Chile (INCC) and by the human research ethics committee of the University of Chile. Patients with histological confirmation of OPSCC, aged 18 years or older, referred to the INCC between the years 2012 and 2023 were included. The information was collected from the database of the head and neck oncology committee, Medical Statistical Organisation Service, ARIA radiotherapy software and clinical record of each patient. The association with HPV was determined by immunohistochemistry for p16 (IHQ p16) being considered as present in the case of having more than 70% positivity [10] (Figure 1). Informed consent was obtained from patients who were alive and could be contacted at the time of the study, according to the guidelines of the ethics and research committee. The date and causes of death were obtained from the centralised data repository of the INCC, which is an information warehouse with ordered data including information from the database of the civil registry of Chile, which is updated weekly [11]. Descriptive statistics with measures of central tendency and proportions were used. For the exploratory analysis of association, the *χ*^2^ test was used for qualitative variables and *t*-student for quantitative variables (parametric), with *p* < 0.05.

## Results

### Description of the population

A total of 178 patients treated during the period studied were analysed. The median age was 61 years (range 37–85 years) and 77% (137) were male. In relation to smoking habits, the median pack-year index (PYI) was 20 (interquartile range = 35). Sixty percent (106) of patients were smokers with a PYI ≥10, and 64% (114) had active alcohol consumption. Of the total, 147 patients were referred from the metropolitan region (MR) and 31 from other regions (Table 1).

### Association with the human papilloma virus

Of the total, 148 patients (83%) had p16 determination, resulting positive in 99 of them, which corresponds to 67% of the sample studied (CI95 59%–74%) during the entire period. An increase in patients with oropharyngeal cancer referred to the INCC stands out, concentrating 61.8% between 2018 and 2023, the same period in which p16 determination begins to be routinely performed. Regarding p16(+) patients, their proportion had a progressive increase from 63% (CI95 45%–78%) in the period 2012–2015 to 77% (CI95 64%–86%) in the period 2020–2023 (Table 2). If we consider the last 2 years of study, the prevalence of p16(+) observed is 85% (22 patients, CI95 72%–94%), Table 2. Regarding the characteristics of the p16(+) population, they corresponded to younger patients and with a lower prevalence of classical risk factors (Table 3).

### Clinical features

The most frequently involved anatomical subsite was the palatine tonsil, with a significantly higher prevalence of p16 (+) compared to other subsites (77%, *p* < 0.037). Most of the patients presented at a loco-regionally advanced stage, with 65% of the sample at stages T3–T4 and 75% at stage N2–N3 according to the American Joint Committee on Cancer TNM staging system (AJCC/TNM) 7th edition classification. Nine patients (5%) had distant metastases at the time of diagnosis. Regarding performance status at admission, 158 patients (89%) were classified as Eastern cooperative oncology group performance status (ECOG) 0–1 (Table 4), with no significant differences regarding the affected subsite (*p* = 0.43) or age group (*p* = 0.64).

Table 5 summarises the prognostic groups according to the AJCC/TNM 8th edition classification.

### Treatment pattern

The initial intention of treatment was curative in 146 (82%) of the patients. In 167 (94%) the primary treatment used was radiotherapy. In all the cases in which the initial treatment was surgery, this was indicated due to the presence of multiple cervical adenopathies (lymph node dissection +/- oropharyngectomy) requiring later adjuvant radiotherapy in all the cases. The technique used in 59% of the patients treated with curative intent was volumetric modulated arc therapy (VMAT). In all cases treated with palliative intent, a conventional 3D technique was used. Eighty-two percent of patients treated with curative intent received concomitant chemotherapy, with a mean dose of 245 mg/m^2^ of cisplatin. Six patients (4%) failed to initiate treatment due to disease progression.

The mean time between biopsy and initiation of oncologic treatment was 3.5 months (range 1–10.7 months), with no significant differences between patients referred from MRI versus other regions (*p* = 0.19).

### Lethality

Regarding the cause of death, in 15 patients (19.48%) this was not related to oropharyngeal cancer (1 non-Hodgkin’s lymphoma, 2 decompensation of diabetes, 1 lung cancer, 1 anaplastic thyroid cancer, 1 oesophageal cancer, 2 acute myocardial infarction, 1 pulmonary fibrosis, 1 terminal renal disease, 1 massive stroke, 4 sepsis: 2 abdominal, 1 urinary and 1 aspiration pneumonia in patient without evidence of oncological disease). Considering the above, the lethality for oropharyngeal cancer in all patients evaluated, in patients treated with curative intent and in those treated with palliative intent was 34.83% (62/178), 23.28% (34/146) and 87.75% (28/32), respectively.

## Discussion

The incidence of oropharyngeal cancer has increased during the last decades, which has been associated with HPV as a pathogenic element, defining a disease with a biological behaviour different from oropharyngeal cancer associated with classic risk factors [3, 6, 7].

A study published in 2021 showed that the prevalence of HPV-related oropharyngeal cancer worldwide was 31%, with a range varying between 0% and 85% depending on the geographic region assessed, being highest in South Korea and Lebanon [12]. Mehanna *et al* [1] showed in a systematic review of patients mainly from North America and Europe that the prevalence of HPV in oropharyngeal cancer increased from 40.5% before 2,000%–72% after 2005.

The data observed in the present study show a similar trend but shifted temporally by 15 years, exceeding 70% prevalence after 2020. The characteristics of the p16(+) patients corresponded to those published internationally [5, 7]. The median age of the population was 58.8 years, with a lower prevalence of alcohol and tobacco consumption.

In South America, Brazil, has been the country that has published the most on the subject. Data have shown that, despite being a country with a high incidence of head and neck cancer, the association with HPV is relatively low, with a reported prevalence of 17.93% [9]. In Colombia, 6/45 (13%) of patients with oropharyngeal cancer recruited between 1999 and 2008 tested positive for HPV by polymerase chain reaction [13].

The INCC population shows a higher prevalence than that published in South America. The differences may be determined by the time period selected in our study (2012–2023), by the detection technique (IHC p16) and by the concentration of urban population (82% of patients from the MR). Oliva *et al* [14] recently published data from a university hospital in Chile, finding positivity for p16 IHC in 28/48 patients (58.3%). Oliva *et al* [14] which is consistent with a 60% prevalence in the INCC population in the period prior to 2020.

Historically, surgical management of locally intermediate or advanced oropharyngeal cancers required large, invasive surgical approaches to achieve a bloc excision with negative margins. These approaches include lingual mandibular release [15]. Transpharyngeal approaches with lateral or suprahyoid pharyngotomies [16, 17]. Pilcher and Zeitels *et al* [16, 17]and transmandibular approaches [18]. Due to the significant morbidity and functional alterations, highlighting the compromise of language and swallowing [19]. Parsons *et al* [19] open surgical approaches lost ground to organ preservation protocols with concurrent chemo-radiotherapy, which became the current standard of treatment [20, 21]. However, organ preservation protocols are not free of complications and morbidity. This is especially important in the group of patients with HPV-associated oropharyngeal cancer, who have longer overall and disease-free survival. Different strategies have been developed with the aim of reducing adverse effects [22] among which are the use of advanced radiotherapy techniques and the use of less invasive surgical techniques such as transoral laser microsurgery [23, 24] or transoral robotic surgery [25] in early stage oropharyngeal cancer. At the international level, there has been a new development of these less invasive techniques [26–28] with good oncologic results in a long series of one centre [29]. Brody *et al* [29] as well as in prospective multi-institutional series [30]. However, the results of the ORATOR2 study, prematurely closed due to G5 toxicity in the surgical arm, show the need for such procedures to be performed by certified surgeons in highly experienced centres [31].

In the present study, 94% of the patients were treated with radiotherapy as the first line of treatment. None of the early stage patients underwent surgery, which is consistent with the lack of access to transoral surgical techniques in the Chilean public system.

Intensity-modulated radiation therapy (IMRT) is internationally considered the standard of treatment to reduce late toxicity. This technique was available at INCC since 2018. For this reason, although in the whole period patients treated with VMAT corresponded to only 59% of the sample, since 2018 most patients were treated with this technique.

As quality criteria in oropharyngeal cancer, it has been established that the time between diagnosis and initiation of treatment should be ≤28 days [32]. In our study, the latency was almost four times longer than recommended, which could explain why 4% of the patients initially considered for curative treatment did not initiate it due to disease progression. This is a consequence of the difficulties related to access and timeliness of treatment in the Chilean public health system, the origin of which is multifactorial and beyond the scope of the present study. In addition, it is important to consider that squamous head and neck cancer in Chile is not one of the malignant neoplasms whose timely management is guaranteed by law [33]. This makes it even more complex in practice to prioritise these cases.

Finally, 19.48% of the observed deaths were not attributable to OPSCC, which is consistent with internationally published data and reflects the high burden of comorbidities present in the group of patients with OPSCC. Kao *et al* [34] analysed the competing causes of death in patients with oropharyngeal cancer in a North American population representative of the period prior to the epidemiologic transition (1988–2001), showing that in 36% of the cases the cause of mortality was not related to OPSCC [34]. This higher proportion, with respect to that observed in our study, may be related to the epidemiological differences between the two populations, the INCC population being representative of the post-epidemiological transition period and therefore including patients with fewer classical risk factors such as tobacco and alcohol, both habits associated with the development of other neoplasms and cardiovascular diseases [35, 36].

The present study has several limitations. On the one hand, it is mainly representative of the urban area of the MR of Santiago, whose epidemiological pattern could differ from more distant regions, so it is important to extend the sample to rural areas far from the capital in order to have a more complete picture at the national level. On the other hand, p16 IHC was used as a method to determine the association with HPV. Although it is the technique suggested by the American College of Pathologists as a screening technique, detection with this technique has a lower specificity compared to the use of *in situ* hybridisation or other molecular techniques, which could affect the final result. Finally, there is a proportion close to 20% of patients whose association with HPV could not be determined, mainly in patients whose diagnosis was made prior to 2016.

## Conclusion

The epidemiological profile in a Chilean public health service, with a mainly urban population, is currently representative of the epidemiological transition that has existed in patients with OPSCC in developed countries in the past. It is important to consider this new epidemiological profile for the development of public health policies and research associated with this topic.

## List of abbreviations

AJCC/TNM, American Joint Committee on Cancer TNM staging system; OPSCC, Squamous cell carcinoma of the oropharynx; HPV, Human papilloma virus; INCC, National Cancer Institute of Chile; IPA, Pack-year index; MR, Metropolitan region of Chile.

## Conflicts of interest

The authors declare that they have no conflicts of interest.

## Funding

Project financed by ANID + FONDEF/XVIII National Competition for Health Research and Development Projects, FONIS SAI21|0003.

## Author contributions

FCV: Recruitment, data collection, analysis, drafting and review. FCM: Data collection, analysis, drafting and review. BPV: Data collection CEO: Data collection, review. TAB: Statistical analysis, writing and review. MAB: Data collection, review. LAM: Data collection, review. FAB: Data collection, writing, review. PAM: Data collection, review. CFA: Data collection, analysis, review. JEO: Analysis, drafting, review. EAV: Data collection, writing. FAF: Data collection. AJDR: Data collection, immunohistochemical analysis. SRI: Data collection, immunohistochemical analysis. EAC: Drafting, review. HRC: Immunohistochemical analysis, Drafting, review.

## Figures and Tables

**Table 1.** Epidemiological characteristics of patients with oropharyngeal cancer treated at the INCC.

Feature	*n* (%)
Origin	
MR	147 (82)
Other regions	31 (18)
Sex	
Male	137 (77)
Female	41 (23)
p16	
(+)	99 (56)
(-)	48 (27)
Unknown	31 (17)
Alcohol	
Present	114 (64)
Absent	64 (36)
Tobacco	
Absent	53 (30)
PYI <10	19 (10)
PYI ≥10	106 (60)

MR: Santiago metropolitan region

**Table 2.** Evolution of p16 prevalence in different periods.

	PERIOD		
	2012-2015	2016-2019	2020-2023
p16+	19 (63%)	36 (60%)	44 (77%)
p16-	11 (37%)	24 (40%)	13 (23%)

**Table 3.** Differences in epidemiological characteristics between p16(+) and p16 (-) patients.

Feature	p16 (+)	p16 (-)	
Male gender	77%	75%	*p* < 0.813
OH (+)	50%	79%	*p* < 0.001
IPA ≥ 10	38%	85%	*p* < 0.0001
Age (Mean, years)	58.8	65.8	*p* < 0.0001

IPA: pack-year index, OH: alcohol consumption

**Table 4.** Clinical characteristics of patients with oropharyngeal cancer treated at the INCC.

Feature	*n* (%)
ECOG	
0	80 (45)
1	78 (44)
2	10 (6)
3	7 (4)
4	2 (1)
Location	
Palatine tonsil	133 (74)
Base of tongue	35 (20)
Soft palate	9 (5)
Posterior pharynx	1 (1)
T	
T1	12 (7)
T2	50 (28)
T3	49 (28)
T4	67 (37)
N	
N0	9 (5)
N1	36 (20)
N2	88 (49)
N3	45 (26)
M	
M0	167 (94)
M1	11 (6)
AJCC TNM 7th edition stage	
I	0
II	3 (2)
III	25 (14)
IVA	96 (54)
IVB	45 (25)
IVC	9 (5)

ECOG: Eastern cooperative oncology group performance status

**Table 5.** Prognostic staging according to AJCC/TNM 8th edition.

Feature	*n* (%)
p16(+)	
I	14 (14)
II	43 (43)
III	38 (38)
IV	4 (4)
p16(-) and unknown	
I	0
II	1 (1)
III	9 (12)
IVA	42 (53)
IVB	22 (28)
IVC	5 (6)

Unknown: There is no record of determination by IHQ p16

## References

[1] Mehanna H, Beech T, Nicholson T (2013). Prevalence of human papillomavirus in oropharyngeal and nonoropharyngeal head and neck cancer – systematic review and meta-analysis of trends by time and region. Head Neck.

[2] Stein AP, Saha S, Yu M (2014). Prevalence of human papillomavirus in oropharyngeal squamous cell carcinoma in the United States across time. Chem Res Toxicol.

[3] Chaturvedi AK, Anderson WF, Lortet-Tieulent J (2013). Worldwide trends in incidence rates for oral cavity and oropharyngeal cancers. J Clin Oncol Off J Am Soc Clin Oncol.

[4] Damgacioglu H, Sonawane K, Zhu Y (2022). Oropharyngeal cancer incidence and mortality trends in all 50 states in the US, 2001-2017. JAMA Otolaryngol Neck Surg.

[5] Gillison ML, D'Souza G, Westra W (2008). Distinct risk factor profiles for human papillomavirus type 16-positive and human papillomavirus type 16-negative head and neck cancers. JNCI J Natl Cancer Inst.

[6] Chaturvedi AK, Freedman ND, Abnet CC (2022). The evolving epidemiology of oral cavity and oropharyngeal cancers. Cancer Res.

[7] Lechner M, Liu J, Masterson L (2022). HPV-associated oropharyngeal cancer: epidemiology, molecular biology and clinical management. Nat Rev Clin Oncol.

[8] Bouvard V, Baan R, Straif K (2009). A review of human carcinogens – part B: biological agents. Lancet Oncol.

[9] Oliveira AC, Cavalcanti de Lima IC, Frez Marques VM (2022). Human papillomavirus prevalence in oral and oropharyngeal squamous cell carcinoma in South America: a systematic review and meta-analysis. Oncol Rev.

[10] Lewis JS, Beadle B, Bishop JA (2018). Human papillomavirus testing in head and neck carcinomas: guideline from the college of American pathologists. Arch Pathol Lab Med.

[11] Floriano F (2023). Methodology for the Construction of a Centralized Data Repository at the National Cancer Institute.

[12] Carlander AF, Jakobsen KK, Bendtsen SK (2021). A contemporary systematic review on repartition of HPV-positivity in oropharyngeal cancer worldwide. Viruses.

[13] Quintero K, Giraldo GA, Uribe ML (2013). Human papillomavirus types in cases of squamous cell carcinoma of head and neck in Colombia. Braz J Otorhinolaryngol.

[14] Oliva C, Carrillo-Beltrán D, Boettiger P (2022). Human papillomavirus detected in oropharyngeal cancers from Chilean subjects. Viruses.

[15] Stanley RB (1984). Mandibular lingual releasing approach to oral and oropharyngeal carcinomas. Laryngoscope.

[16] Pilcher LS (1886). I. On lateral pharyngotomy for the extirpation of malignant tumors of the tonsillar region. Ann Surg.

[17] Zeitels SM, Vaughan CW, Ruh S (1991). Suprahyoid pharyngotomy for oropharynx cancer including the tongue base. Arch Otolaryngol Head Neck Surg.

[18] Christopoulos E, Carrau R, Segas J (1992). Transmandibular approaches to the oral cavity and oropharynx. A functional assessment. Arch Otolaryngol Head Neck Surg.

[19] Parsons JT, Mendenhall WM, Stringer SP (2002). Squamous cell carcinoma of the oropharynx: surgery, radiation therapy, or both. Cancer.

[20] Calais G, Alfonsi M, Bardet E (1999). Randomized trial of radiation therapy versus concomitant chemotherapy and radiation therapy for advanced-stage oropharynx carcinoma. J Natl Cancer Inst.

[21] Blanchard P, Landais C, Petit C (2016). Meta-analysis of chemotherapy in head and neck cancer (MACH-NC): an update on 100 randomized trials and 19,248 patients, on behalf of MACH-NC group. Ann Oncol.

[22] Ansinelli H, Gay C, Nguyen S (2023). Personalized precision radiotherapy and its evolving role for human papillomavirus-positive oropharyngeal cancer. J Natl Cancer Center.

[23] Canis M, Martin A, Kron M (2013). Results of transoral laser microsurgery in 102 patients with squamous cell carcinoma of the tonsil. Eur Arch Otorhinolaryngol.

[24] Canis M, Ihler F, Wolff HA (2013). Oncologic and functional results after transoral laser microsurgery of tongue base carcinoma. Eur Arch Otorhinolaryngol.

[25] O'Malley BW, Weinstein GS, Snyder W (2006). Transoral robotic surgery (TORS) for base of tongue neoplasms. Laryngoscope.

[26] Mirghani H, Blanchard P (2017). Treatment de-escalation for HPV-driven oropharyngeal cancer: where do we stand?. Clin Transl Radiat Oncol.

[27] de Almeida JR, Li R, Magnuson JS (2015). Oncologic outcomes after transoral robotic surgery. JAMA Otolaryngol Head Neck Surg.

[28] Arens C (2012). Transoral treatment strategies for head and neck tumors. GMS Curr Top Otorhinolaryngol Head Neck Surg.

[29] Brody RM, Shimunov D, Cohen RB (2022). A benchmark for oncologic outcomes and model for lethal recurrence risk after transoral robotic resection of HPV-related oropharyngeal cancers. Oral Oncol.

[30] Ferris RL, Flamand Y, Weinstein GS (2022). Phase II randomized trial of transoral surgery and low-dose intensity modulated radiation therapy in resectable p16+ locally advanced oropharynx cancer: an ECOG-ACRIN cancer research group trial (E3311). J Clin Oncol Off J Am Soc Clin Oncol.

[31] Palma DA, Prisman E, Berthelet E (2022). Assessment of toxic effects and survival in treatment de-escalation with radiotherapy versus transoral surgery for HPV-associated oropharyngeal squamous cell carcinoma: the ORATOR2 phase 2 randomized clinical trial. JAMA Oncol.

[32] Katsoulakis E, Kudner R, Chapman C (2022). Consensus quality measures and dose constraints for head and neck cancer with an emphasis on oropharyngeal and laryngeal cancer from the veterans affairs radiation oncology quality surveillance program and American Society for radiation oncology expert panel. Pract Radiat Oncol.

[33] Orientation in Health (2023). Superintendencia de Salud, Government of Chile.

[34] Kao J, Lau KHV, Tong CCL (2012). Competing causes of death in patients with oropharyngeal cancer treated with radiotherapy. Exp Ther Med.

[35] Corrao G, Bagnardi V, Zambon A (2004). A meta-analysis of alcohol consumption and the risk of 15 diseases. Prev Med.

[36] Iribarren C, Tekawa IS, Sidney S (1999). Effect of cigar smoking on the risk of cardiovascular disease, chronic obstructive pulmonary disease, and cancer in men. N Engl J Med.

